# Supplementary material for: SIP2 is the master transcription factor of Plasmodium merozoite formation
Source: Sci Adv. 2025 Mar 21;11(12):eads5458. doi: 10.1126/sciadv.ads5458 (PMC11927625; doi:10.1126/sciadv.ads5458)
Supplement: Supplementary file 1 — Figs. S1 to S4 Tables S1 to S4 Legends for data S1 to S4 [file sciadv.ads5458_sm.pdf]

Science Advances
AAAS

Supplementary Materials for

SIP2 is the master transcription factor of *Plasmodium* merozoite formation

Tsubasa Nishi *et al.*

Corresponding author: Masao Yuda, m-yuda@med.mie-u.ac.jp

Sci. Adv. **11**, eads5458 (2025)
DOI: 10.1126/sciadv.ads5458

The PDF file includes:

Figs. S1 to S4
Tables S1 to S4
Legends for data S1 to S4

Other Supplementary Material for this manuscript includes the following:

Data S1 to S4

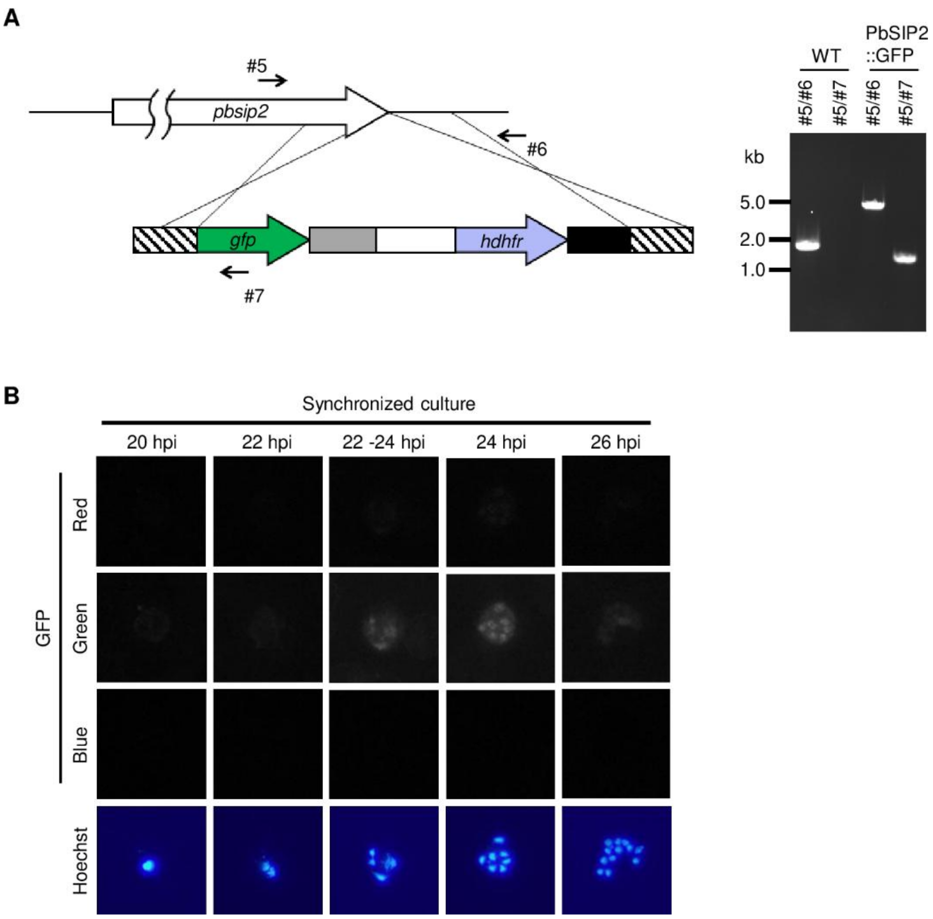

Fig. S1. PbSIP2 expression during asexual-blood stage development.

(A) Genotyping of PbSIP2::GFP. Schematic illustration of gene editing at the *pbsip2* locus is shown on the left. A gel image from the genotyping PCR analysis is shown on the right side. The primer numbers are listed in Data S4. (B) Expression of PbSIP2 in schizonts of the PbSIP2::GFP. The GFP signals are shown as grey scale images for each RGB channel. Nuclei were stained with Hoechst 33342. Scale bar = 5 μm

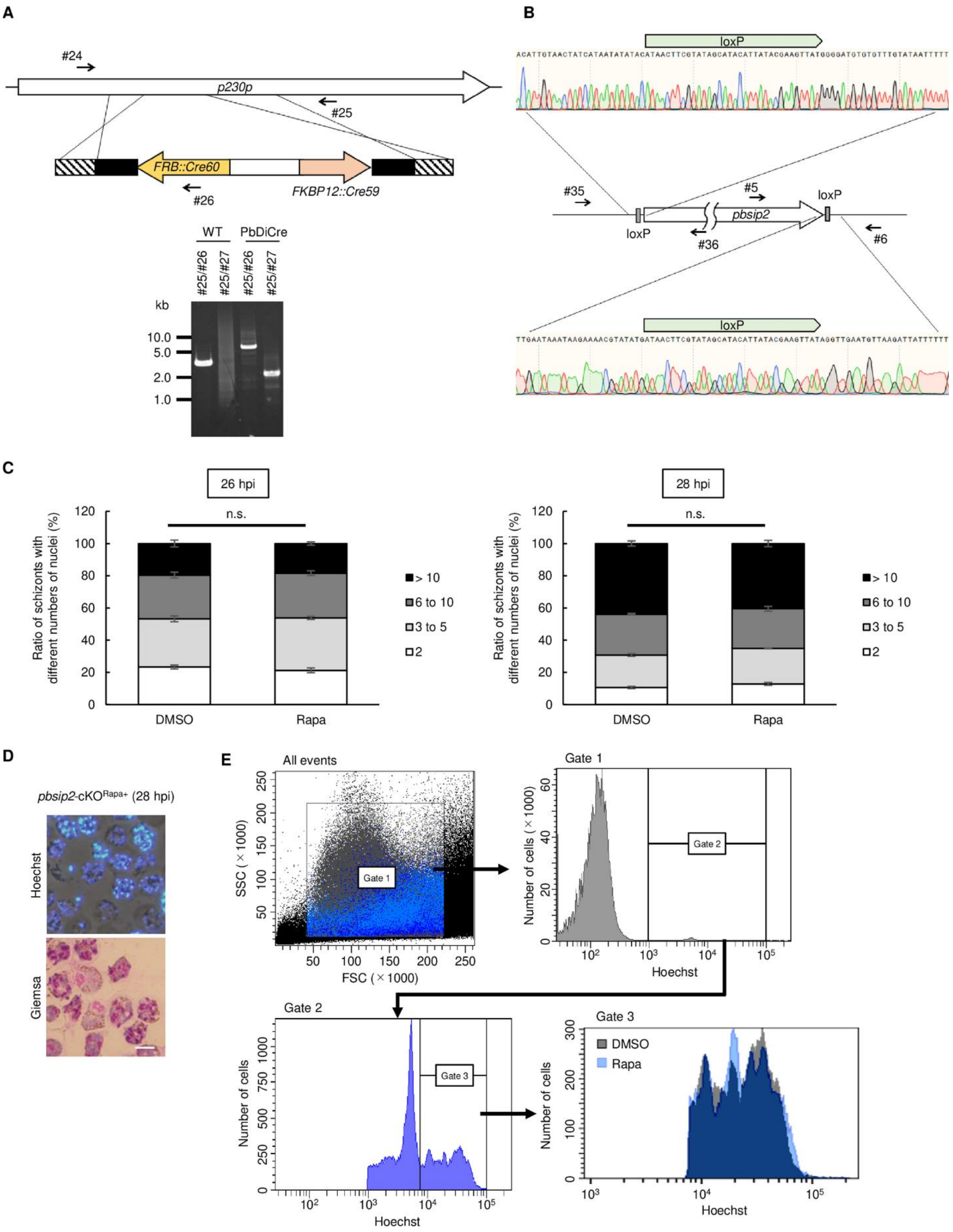

Fig. S2. DiCre-mediated conditional knockout of *pbsip2*.

(A) Genotyping of PbDiCre. Schematic illustration of gene editing at the *p230p* locus is shown at the top. A gel image of genotyping PCR analysis is shown at the bottom. The primer numbers are listed in Data S4. (B) Genotyping of *pbsip2*-cKO. A schematic illustration of the *pbsip2* locus in *pbsip2*-cKO is shown. Sangar sequence results confirming insertion of loxP at 5'- and 3'-side of *pbsip2* are shown on the top and bottom, respectively. (C) Ratio of schizonts with different numbers of nuclei for PbDiCre in the absence and presence of rapamycin at 26 hpi (left) and 28 hpi (right). Error bars indicate the standard error of the mean values from three independent experiments. The *p*-values were calculated using a two-tailed Student's t-test. (D) Representative images of *pbsip2*-cKO^{Rapa+} at 28 hpi. A Hoechst-stained fluorescence image merged with a phase contrast microscopic image (top) and a Giemsa-stained image (bottom) are shown. Scale bar = 5 μm. (E) Gating strategies for the flow cytometric analysis presented in Fig. 2G. Representative images from the analysis using *pbsip2*-cKO^{Rapa-} are shown. The panel for all events shows side scatter (SSC) vs forward scatter (FSC) density plot, and Gate 1, 2, and 3 panels show histogram of Hoechst intensity. For Gate 3, representative images for both *pbsip2*-cKO^{Rapa-} and *pbsip2*-cKO^{Rapa+} are shown. Mean Hoechst intensities in Fig 2G were derived from the Gate 3 population, in which mononuclear parasites were excluded. Nuclei were stained with Hoechst 33342.

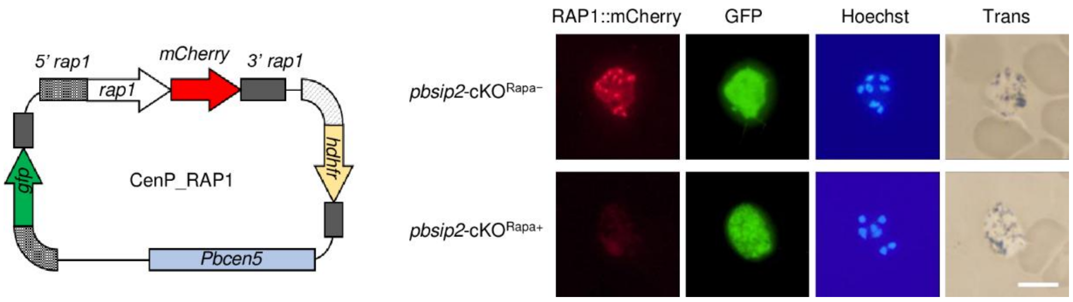

Fig. S3.

Episomal expression of RAP1 fused with mCherry in *pbsip2*-cKO. On the left, schematic illustration of CenP_RAP1 is shown. Pbcen5 indicates the centromere sequence of *P. berghei* chromosome 5. On the right, representative images of schizonts for *pbsip2*-cKO^{Rapa-} and *pbsip2*-cKO^{Rapa+} with CenP_RAP1 are shown. GFP was expressed under the control of *pbhsp70* promoter as an internal control. At least 50 GFP-positive schizonts were assessed for the mCherry-fused RAP1 expression. Nuclei were stained with Hoechst 33342. Scale bar = 5 µm.

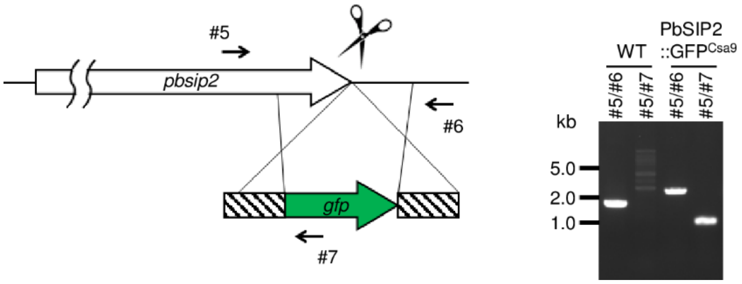

Fig. S4.

Genotyping of PbSIP2::GFP^{Cas9}. Schematic illustration of gene editing at the *pbsip2* locus is shown on the left. A gel image from the genotyping PCR analysis is shown on the right side. The primer numbers are listed in Data S4.

Table S1.

(A) Ratio of schizonts with different numbers of nuclei for PbDiCre in the absence and presence of rapamycin at 26 hpi and 28 hpi (%).

	Num. of nuclei	Rapa−			Rapa+		
		1	2	3	1	2	3
26 hpi	> 10	18.0	17.0	23.8	20.4	17.1	17.7
	6 to 10	30.0	27.7	23.8	30.6	27.0	25.7
	3 to 5	30.0	33.0	26.7	30.6	33.3	33.6
	2	22.0	22.3	25.7	18.4	22.5	23.0
28 hpi	> 10	44.8	46.0	40.7	37.4	39.8	44.2
	6 to 10	25.0	24.8	26.3	26.0	26.2	21.7
	3 to 5	20.8	18.6	21.2	22.0	22.3	21.7
	2	9.4	10.6	11.9	14.6	11.7	12.3

(B) Ratio of schizonts with different numbers of nuclei for *pbsip2*-cKO in the absence and presence of rapamycin at 26 hpi and 28 hpi (%).

	Num. of nuclei	Rapa−			Rapa+		
		1	2	3	1	2	3
26 hpi	> 10	21.4	21.9	15.7	0	1.1	2.1
	6 to 10	22.4	27.6	20.6	45.7	44.3	45.4
	3 to 5	31.6	28.6	40.2	30.9	27.3	23.7
	2	24.5	21.9	23.5	23.5	27.3	28.9
28 hpi	> 10	47.6	39.1	48.4	2.1	1.8	0
	6 to 10	28.2	29.6	30.8	70.1	63.4	75.3
	3 to 5	17.5	19.1	15.4	22.7	21.4	17.2
	2	6.8	12.2	5.5	5.2	13.4	7.5

(C) Ratio of schizonts with different numbers of Hoechst foci for *pbsip2*-cKO in the absence and presence of rapamycin at 28 hpi (%).

Num. of Hoechst foci	Rapa−			Rapa+		
	1	2	3	1	2	3
> 10	64.7	61.3	54.2	38.7	48.3	40.4
6 to 10	26.9	29.6	33.3	43.2	40.0	45.7
3 to 5	6.0	7.0	9.0	11.7	9.0	8.6
2	2.4	2.1	3.5	6.3	2.8	5.3

Table S2.

Gene ontology analysis for the PbSIP2 targets. Terms with a *p*-value less than 0.01 are shown. Inf in odds ratio column indicates an infinite number.

Term	*p*-value	Odds ratio
apical part of cell	1.96E-21	7.95
apical complex	3.85E-18	8.92
rhoptry	1.50E-16	20.68
rhoptry neck	1.35E-10	Inf
protein binding	8.86E-07	2.19
nucleosome	9.38E-07	61.57
movement in host	1.85E-06	4.91
pellicle	2.69E-06	4.44
protein heterodimerization activity	2.88E-06	17.10
biological process involved in symbiotic interaction	4.52E-06	4.30
biological process involved in interaction with host	4.52E-06	4.30
inner membrane pellicle complex	5.08E-06	4.42
biological process involved in interspecies interaction between organisms	6.56E-06	4.16
entry into host	2.25E-05	5.00
host cell cytoplasm part	4.75E-05	3.09
host intracellular part	5.08E-05	2.83
host cell cytoplasm	5.08E-05	2.83
host intracellular region	6.02E-05	2.80
mitochondrial respirasome	3.52E-04	6.39
transmembrane transporter complex	3.67E-04	10.49
host cell part	3.72E-04	2.43
basal part of cell	5.43E-04	34.71
chromatin	7.87E-04	5.40
mitochondrial respiratory chain complex III	1.08E-03	10.88
respiratory chain complex III	1.08E-03	10.88
protein-DNA complex	1.13E-03	4.40
transporter complex	1.14E-03	7.48
chromosome	1.24E-03	2.43
protein dimerization activity	1.66E-03	4.47
calcium ion binding	2.06E-03	3.83

cytochrome complex	2.15E-03	4.38
inner mitochondrial membrane protein complex	2.15E-03	4.38
host cell surface binding	2.44E-03	7.51
host cell	2.79E-03	2.01
host cellular component	2.79E-03	2.01
symbiont-containing vacuole	3.02E-03	2.56
extracellular membrane-bounded organelle	3.02E-03	2.56
extracellular organelle	3.52E-03	2.51
extracellular region	4.08E-03	2.47
protein kinase C-activating G protein-coupled receptor signaling pathway	4.74E-03	Inf
respiratory chain complex	6.26E-03	3.50
chromosomal region	6.57E-03	2.93
folic acid transmembrane transporter activity	7.80E-03	Inf
ATP-dependent diacylglycerol kinase activity	7.80E-03	Inf
protein phosphorylation	8.16E-03	2.48
phosphorylation	9.01E-03	2.44
cell surface	9.06E-03	2.29
cell periphery	9.21E-03	2.21
exit from host cell	9.74E-03	4.63
chromosome, centromeric region	9.80E-03	3.18

Table S3.
Gene ontology analysis for the genes significantly downregulated in *pbsip2*-cKO^{Rapa+} compared with *pbsip2*-cKO$^{Rapa−}$. Terms with a *p*-value less than 0.01 are shown. Inf in odds ratio column indicates an infinite number.

Term	*p*-value	Odds ratio
apical part of cell	6.64E-36	25.26
apical complex	4.83E-31	27.69
rhoptry	4.36E-26	62.61
rhoptry neck	1.78E-14	Inf
biological process involved in symbiotic interaction	2.49E-10	12.37
biological process involved in interaction with host	2.49E-10	12.37
biological process involved in interspecies interaction between organisms	3.59E-10	11.99
movement in host	4.04E-09	11.98
entry into host	2.25E-07	11.69
host cell surface binding	6.35E-07	33.39
pellicle	6.60E-07	7.07
inner membrane pellicle complex	2.52E-06	6.76
host cell cytoplasm part	1.57E-05	4.69
symbiont-containing vacuole	2.21E-05	5.25
extracellular membrane-bounded organelle	2.21E-05	5.25
extracellular organelle	2.59E-05	5.16
cell surface	2.59E-05	5.16
host cell surface receptor binding	2.96E-05	43.29
host cell	3.00E-05	3.60
host cellular component	3.00E-05	3.60
extracellular region	3.02E-05	5.06
calcium ion binding	6.30E-05	8.70
cell motility	7.65E-05	8.42
exoneme	8.37E-05	Inf
host intracellular part	1.53E-04	3.68
host cell cytoplasm	1.53E-04	3.68
host intracellular region	1.70E-04	3.64
host cell part	5.30E-04	3.21
protein phosphorylation	7.00E-04	4.90
phosphorylation	7.61E-04	4.83

PTEX complex	7.84E-04	33.47
catalytic activity, acting on a protein	7.86E-04	2.54
exit from host cell	1.27E-03	10.49
translocation of molecules into host	1.83E-03	80.91
translocation of peptides or proteins into host cell cytoplasm	1.83E-03	80.91
translocation of peptides or proteins into host	1.83E-03	80.91
rhoptry membrane	1.93E-03	Inf
microneme	2.27E-03	5.26
myosin complex	2.57E-03	16.72
protein kinase activity	2.85E-03	3.77
phosphotransferase activity, alcohol group as acceptor	3.07E-03	3.40
protein binding	5.92E-03	1.99
symbiont-containing vacuole membrane	6.63E-03	4.91
kinase activity	8.56E-03	2.69
phospholipid transporter activity	9.22E-03	21.03
iron ion binding	9.22E-03	21.03
protein modification process	9.43E-03	2.50

Table S4.

(A) %input values in the ChIP-qPCR analysis of PbSIP2 using PbSIP2::GFP^{Cas9} and PbSIP2::GFP^{Cas9_cismut}. [*rop14*: PBANKA_0111600, *ralp1*: PBANKA_0619700, *ron4*: PBANKA_0932000, *trap*: PBANKA_1349800]

Gene	PbSIP2::GFP^{Cas9}			PbSIP2::GFP^{Cas9_cismut}		
	1	2	3	1	2	3
rop14	0.15	0.20	0.19	0.015	0.011	8.7E-3
ralp1	0.19	0.18	0.15	0.15	0.21	0.10
ron4	0.15	0.16	0.077	0.12	0.10	0.086
trap	7.2E-3	5.3E-3	6.6E-3	5.4E-3	7.4E-3	3.5E-3

(B) Relative expression of *rop14*/*ralp1* in the RT-qPCR analysis using PbSIP2::GFP^{Cas9} and PbSIP2::GFP^{Cas9_cismut}.

Parasite	1	2	3
PbSIP2::GFP^{Cas9}	0.58	0.55	0.60
PbSIP2::GFP^{Cas9_cismut}	0.020	0.018	0.021

Data S1. (separate file)

ChIP-seq analysis of PbSIP2. (A) Peaks identified in experiment 1. (B) Peaks identified in experiment 2. (C) Target genes of PbSIP2.

Data S2. (separate file)

Differential expression analysis between *pbsip2*-cKO^{Rapa-} and *pbsip2*-cKO^{Rapa+}.

Data S3. (separate file)

DIP-seq analysis using MBP::PbSIP2.

Data S4. (separate file)

List of primers used in this study.
